# Melatonin-mediated development and abiotic stress tolerance in plants

**DOI:** 10.3389/fpls.2023.1100827

**Published:** 2023-01-26

**Authors:** Yue Pan, Xiaoshan Xu, Lei Li, Qinglin Sun, Qiguang Wang, Huahong Huang, Zaikang Tong, Junhong Zhang

**Affiliations:** ^1^ State Key Laboratory of Subtropical Silviculture, School of Forestry and Biotechnology, Zhejiang A&F University, Hangzhou, Zhejiang, China; ^2^ Hunan Academy of Forestry, Changsha, Hunan, China

**Keywords:** melatonin, abiotic stress, growth and development, stress tolerance, secondary metabolites

## Abstract

Melatonin is a multifunctional molecule that has been widely discovered in most plants. An increasing number of studies have shown that melatonin plays essential roles in plant growth and stress tolerance. It has been extensively applied to alleviate the harmful effects of abiotic stresses. In view of its role in regulating aspects of plant growth and development, we ponder and summarize the scientific discoveries about seed germination, root development, flowering, fruit maturation, and senescence. Under abiotic and biotic stresses, melatonin brings together many pathways to increase access to treatments for the symptoms of plants and to counteract the negative effects. It has the capacity to tackle regulation of the redox, plant hormone networks, and endogenous melatonin. Furthermore, the expression levels of several genes and the contents of diverse secondary metabolites, such as polyphenols, terpenoids, and alkaloids, were significantly altered. In this review, we intend to examine the actions of melatonin in plants from a broader perspective, explore the range of its physiological functions, and analyze the relationship between melatonin and other metabolites and metabolic pathways.

## Introduction

Melatonin (N-acetyl-5-methoxytryptamine), a bioactive molecule, is pervasive in organisms and has been well studied since it was first discovered in the pineal gland of cows (Lerner et al., 1958). Previous studies have demonstrated that melatonin is essential for maintaining human circadian rhythm, sleep, mood, body temperature, appetite, and immunological responses (Socaciu et al., 2020). Furthermore, antioxidant, anti-inflammatory, and other biological functions were also proven to be regulated by melatonin; thus, melatonin has been employed extensively for disease pathogenesis and drug development (Millet-Boureima et al., 2021).

A previous review on melatonin has provided insight into the biosynthesis, catabolism, and physiological and biochemical functions of this important molecule. The biosynthetic and metabolic pathways of melatonin, including tryptophan decarboxylase (*TDC*), tryptamine 5-hydroxylase (*T5H*), serotonin N-acetyltransferase (*SNAT*), N-acetylserotonin methyltransferase (*ASMT*), caffeic-O-methyltransferase (*COMT*), tryptophan hydroxylase (*TPH*), and hydroxyindole-O-methyltransferase (*HIOMT*), have been fully described by Zeng et al. (2022) and Kyoungwhan (2021). It is universally acknowledged that melatonin functions affect all aspects of the plant life cycle from seed germination to growth, maturation, and aging as well as aiding stressed plants in recovery (Zhang et al., 2022). Currently, scientists have elucidated the mechanisms by which melatonin alleviates stress by its function in antioxidants, photosynthesis, ion regulation, and stress signaling. Those basic roles of melatonin suggest that it could be an efficient method to ensure sustainable crop production and food safety. Here we review the myriad roles of melatonin and its possible molecular mechanisms integral to acclimatizing plants to climate action and self-consumption in times of stress. We examine the mechanisms of melatonin activities that underpin plant growth under various conditions and their effect on metabolic pathways and metabolites in response to stress.

## The roles of melatonin in plant growth and development

**Figure 1:**
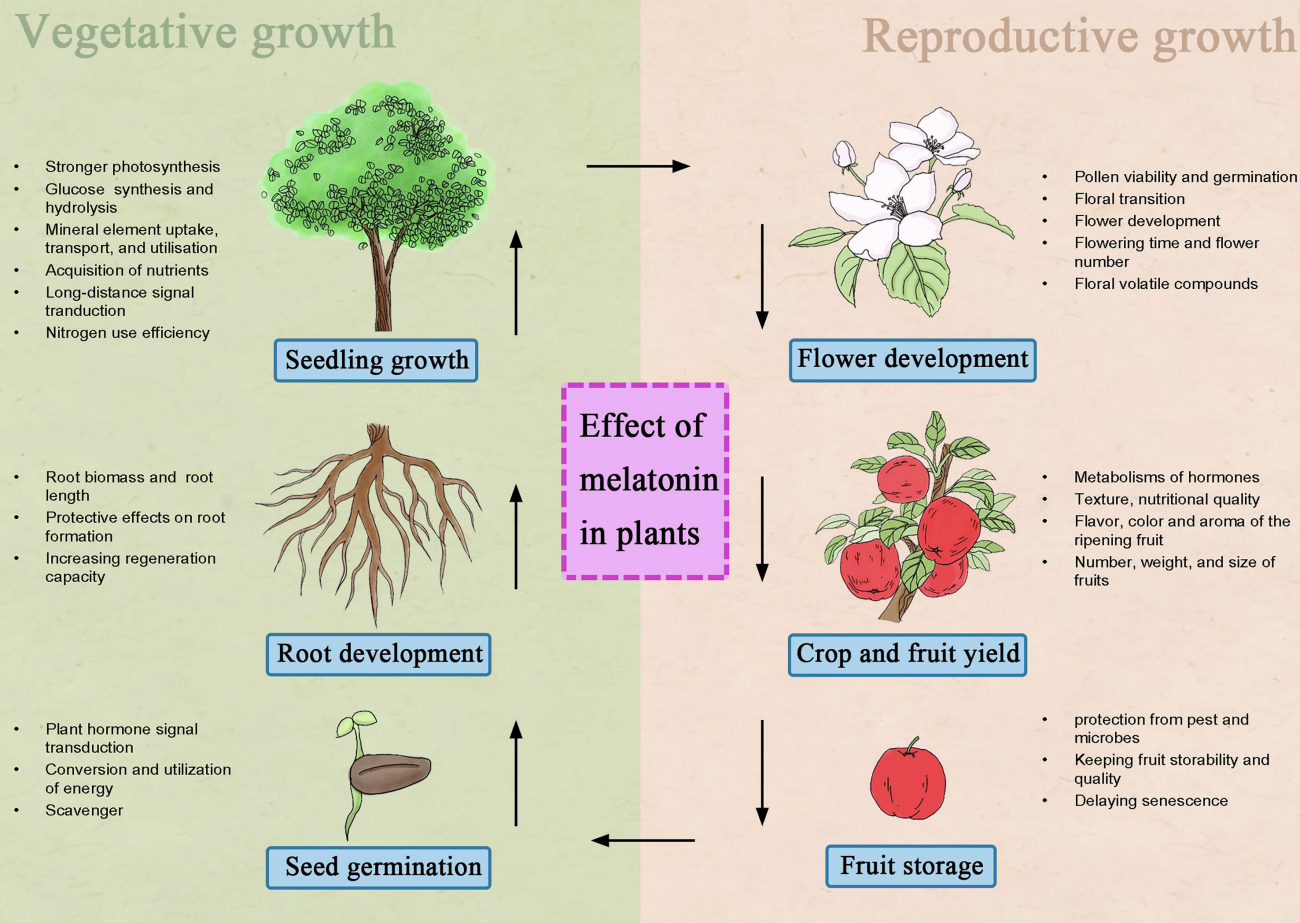
Summary of functional melatonin in plant vegetative growth and reproductive growth.

An increasing number of studies have shown that melatonin has significant effects on growth and development, triggered by dramatic changes in metabolic status and diverse molecular and cellular processes (Wang et al., 2022a; Xie et al., 2022). The role of melatonin in plant life is shown in **Figure 1**.

### Effect of melatonin on seed germination

Seed germination is the initial stage in the plant life cycle and involves the transportation of a series of signaling molecules and an array of gene expression changes through multiple complex physiological processes (Lee and Back, 2022). The crucial role of melatonin in stimulating seed germination was described by Zhang et al. (2022) and Wang et al. (2022), who reported that cell division, shoot initiation, and seed dormancy of *Arabidopsis*, cucumber (*Cucumis sativus*), bermudagrass (*Cynodon dactylon)*, pepper (*Capsicum annuum*), and sweet corn were modulated by melatonin. Melatonin cooperates with hormones, such as indole-3-acetic acid (IAA), abscisic acid (ABA), gibberellin (GA), cytokinins (CKs), and salicylic acid (SA) (Yin et al., 2022)—for example, melatonin improves the germination rate of seeds by mediating changes in endogenous GA and ABA. Upregulating ABA catabolic genes (*CYP707As* and 8′hydroxylase genes) and GA biosynthesis genes (*GA20ox* and *GA3ox*) and downregulating ABA biosynthesis genes (*NCEDs*, *LpZEP*, and *LpNCED1*) contribute to improving hydrolytic enzyme activities (such as α-AMS and β-GAL) and provide the energy needed for seed germination and thus against germination constraints engendered by seed coat limits and embryo dormancy (Chen et al., 2021; Wang et al., 2022a). Genes belonging to the cytokinin-mediated signaling pathway (*AtAHK2* and *AtARRs*) are also upregulated to promote cell division and shoot initiation in the presence of melatonin (Suzuki et al., 2002). The above-mentioned results imply that melatonin has multiple functions in the fine-tuning of hormone homeostasis and signaling in plants. Conversely, it should be noted that melatonin concentrations used to regulate seed germination are highly divergent among species, and its cooperation with ABA, GA, and auxin may impact sprout suppression (Dong D. et al., 2021; Yin et al., 2022). Furthermore, seeds coated with melatonin before sowing had improved germination rate and seed viability, which are specific to melatonin as an antioxidant to remove excess reactive oxygen species (ROS) accumulation and enhance the production capabilities of soluble sugars (Shi et al., 2015a; Wei et al., 2015; Teng et al., 2022). Under salt stress, promoting the synthesis of both new proteins and secondary metabolites is a key mechanism by which melatonin improves the seed germination rate in cotton (*Gossypium hirsutum*) (Chen et al., 2021), tomato (*Solanum lycopersicum*) (Xie et al., 2022), and cucumber (Zhang et al., 2017). In addition, melatonin upregulates protein levels, which are involved not only in stress tolerance but also in cell elongation, glycolysis, citric acid cycle, and glyoxylate cycle (Zhang et al., 2017). Therefore, melatonin works on seed germination partly by promoting energy production and invoking cellular processes and primary and secondary metabolism.

### Regulation of melatonin on root development

Previous studies support the point that melatonin positively affects root developmental regulation (Park and Back, 2012; Mao et al., 2020). Larger root biomass and longer seminal root length are observed in many melatonin-treated plants, such as rice, tomato, and apple (*Malus prunifolia*) (Chen et al., 2019; Mao et al., 2020). Park and Back (2012) demonstrated the involvement of melatonin in an intervention for lateral root (LR) formation. Melatonin regulates the expression levels of the cell cycle-related genes *SlCDKA1*, *SlCYCD3;1*, and *SlKRP2* by stimulating SlPAO1-H_2_O_2_ (polyamine oxidase) and SlRboh3/4-O_2_•^−^ (respiratory burst oxidase homolog), leading to the initial development of lateral root primordia. Mao et al. (2020) showed that melatonin also promotes adventitious root formation. Additionally, the developmental process and correlations between melatonin and hormones (IAA, ABA, GA, and zeatin ribosid) are crucial for AR development—for instance, apple root formation is related to the melatonin-induced expression of WUSCHEL-RELATED HOMEOBOX GENE 11 (*WOX11*) along with IAA synthesis (Wang et al., 2022). However, the crosstalk is intricate—for example, melatonin impedes taproot growth regardless of its concentration in monocot canary grass (*Phalaris canariensis*) and oat (*Avena sativa*) (Wang et al., 2022a). Moreover, the advance of root development by melatonin is the discovery of novel signaling networks consisting of H_2_O_2_, NO, G protein, ROS, and Ca (Chen et al., 2019; Hu C. Z. et al., 2020). In melon (*Cucumis melo*), *A. thaliana*, and alfalfa (*Medicago sativa*), signal transduction provides deep insights for understanding the protective effects of exogenous and endogenous melatonin on LR formation in response to external stimuli (Hu Z. C. et al., 2020; Wang et al., 2022a).

Melatonin induces young branches, cuttings, calli, and *in vitro* cultures of sweet cherry and pomegranates to root (Wang et al., 2022a). Melatonin regulates the physiological and biochemical processes of callus regeneration and improves the differentiation of embryogenic cells, thus improving the regeneration rate under drought stress conditions (Zhou et al., 2022). Taken together, the fluctuation of endogenous melatonin contents may be a candidate determinant to reveal the seasonal effect on the tissue culture response and regeneration frequency of barley explants, which needs further study (Yang S. J. et al., 2022).

### Various roles of melatonin in plant growth

Melatonin plays an indispensable role in plant growth. The growth, root yield, and sugar content of sugar beet seedlings were promoted when melatonin was applied (Zhang et al., 2021). Zhong et al. (2020) found that grape seedlings grew better when melatonin was applied by activating sucrose-decomposing enzymes and sucrose phosphate synthase to hydrolyze and synthesize sucrose as well as the transport of sucrose to nonphotosynthetic “sink” tissues (*e*.*g*., flowers, fruits, seeds, and roots). Furthermore, the regulation of melatonin in its own biosynthesis and degradation promotes specific mineral nutrition signal transduction, and within relatively stable ratios, it can balance the acquisition and appropriate of mineral nutrients, thus improving plant growth (Bawa et al., 2020; Sun et al., 2022). Regardless of whether nutrients (K, Fe, N, and S) are lacking or there is an excess of nutrients (N, Zn, Cu, and AI), there are detectable signs of optimization after melatonin application (Sun et al., 2022). Moreover, positive effects of external melatonin treatments under abiotic stress on seedling growth parameters and plant mineral contents (P, K, Ca, Mg, Fe, Zn, Cu, and Mn) were also observed by Bawa et al. (2020) and Sezer et al. (2021)—for example, when exposed to saline soil conditions, the exogenous application of melatonin to seedlings restores nutrient concentrations (Na^+^, Ca^2+^, and K^+^) and significantly increases Cu^2+^, Mn^2+^, and Zn^2+^ contents (Sezer et al., 2021).

Additionally, melatonin has been considered a circadian oscillator that affects the rhythm of the biological clock system and some physiological indicators in both mammals and plants (Chang et al., 2021). In the majority of plants (*e*.*g*., *A. thaliana*, rice, and barley), the melatonin concentrations are high at night (Chang et al., 2021; Ahn et al., 2021). Melatonin biosynthesis genes (*TDC*, *T5H*, *SNAT*, and particularly *ASMT*) are the core factors in adjusting the circadian clock of plant growth (Ahn et al., 2021). Consisting of light-dependent processes, melatonin production has regulatory relationships with some photoreceptors (such as phytochromes), whose deficiency will influence the gene expression duration of melatonin biosynthesis (Ahn et al., 2021). It is also worth noting that exogenous melatonin application restores the rhythmic expression of core circadian clock genes, while the absence of *GIGANTEA* genes leads to the non-rhythmic expression of *ASMT*, implying a potential melatonin-mediated signaling network (Chang et al., 2021; Ahn et al., 2021).

Additionally, melatonin influences plant growth by regulating the nitrogen (N) metabolism pathways. The nitrate transporter gene *OsNPF6.5*, glutamine synthetase gene *OsGS2*, and amino acid transporter gene *OsAAP14* involved in N metabolism are induced in response to melatonin, and hence tiller number, nitrate uptake, and N use efficiency are affected (Wang et al., 2020). Melatonin regulates relevant key enzymes for N absorption and metabolism (such as S-nitrosoglutathione reductase and nitrate reductase) that are integral to triggering NO accumulation and its feedback (Zhang et al., 2022; Wang et al., 2022a). These valuable hints allow us to further appreciate the contribution of melatonin to plant growth and development, including but not limited to roots, stems, and leaves, which are apt to absorb nutrients and compete for resources.

### The improvement of melatonin in plant reproductive growth

Melatonin has multiple functions in flowering. Melatonin is involved in distinct flowering pathways, including ambient temperature, vernalization, photoperiod, autonomous, GA, and age pathways (Wang et al., 2022a). In the autonomous pathway, it has been indicated that the effect of melatonin requires Flowering Locus C, which implies an innovative pathway in regulating floral transition in *A. thaliana*, with interconnectivity between melatonin and strigolactone (Yang et al., 2019). In GA pathways, the stabilization of transcriptional regulator DELLA proteins mediated by melatonin results in delayed translation (Yang et al., 2019; Wang et al., 2022a). Murch et al. (2009) and Lee and Back, (2019) found that the melatonin biosynthesis gene *SNAT2* is significantly induced in *Arabidopsis* flowers and achieves the highest content in reproductive organs but gradually decreases as flowers mature. Fluctuations in melatonin levels also induce flowering delays, as shown in some algae and photoperiodic plants. In *Antirrhinum majus*, the number, size, and quality of flowers are improved by different concentrations of melatonin (Xiang et al., 2020). Furthermore, it is argued that melatonin influences volatile synthesis gene expression (such as *TPS*, *DXS*, *BSMT*, *HAT*, *GGPS*, and *PAL* in the terpenoid and benzenoid/phenylpropanoid pathways), thus regulating floral volatile compound content (Farhat et al., 2021).

Melatonin reverses the inhibitory effect of stress on pollen viability and germination (Hu W. et al., 2020). This might be attributed to exogenous melatonin at an appropriate dose, thus improving carbohydrate transport into the anthers (Hu et al., 2022). Melatonin promotes the transport of carbon assimilate from leaves to sink tissues by inducing the expression of sucrose transporters (*SUT1* and *SUT2*) related to sucrose phloem loading under drought stress (Hu et al., 2022). Conversely, melatonin maintains carbohydrate metabolism in male and female tissues by accelerating sucrose decomposition and improving the availability of carbohydrates (Hu W. et al., 2020). In cotton anthers, the addition of 100 and 200 μM melatonin markedly stimulates the activities of the main rate-limiting enzymes of starch biosynthesis (AGPase and SSSase), and SuSy, cell wall, and vacuolar invertase activities are also elevated in wheat and tomato (Wang et al., 2022a). Further research revealed that melatonin contributes to higher male fertility of crops by increasing autophagy-related gene expression and autophagosome formation to restore the stability of tapetum cells under high temperature conditions (Qi et al., 2018). Similarly, Nguyen et al. (2010) also showed that melatonin regulates the tricarboxylic acid cycle to meet the energy demand under negative environmental conditions. As mentioned above, rather than being irretrievable, the losses of flower and crop yields recover due to melatonin regulation in multiple fields.

### Melatonin regulates fruit maturation and post-management

#### Fruiting period and quality

Melatonin plays a role in the complicated process of fruit ripening, production, and quality (Wang et al., 2022). This stage is characterized by remarkable changes in the aroma, color, and flavor of the ripening fruit. Compared with control plants, the contents of organic acids, phenolics, flavonoids, peonidin derivatives, and apoptotic inhibitor proteins increased with melatonin treatment (Sun et al., 2016; Xie et al., 2022). Simultaneously, melatonin influences the expression of sucrose invertase genes and the net photosynthetic rate, thus increasing the solute content (*e*.*g*., soluble sugars) and pigment content (anthocyanins and carotenoids) in tomato, pear (*Pyrus communis*), and grape (*Vitis labruscana*) (Wang et al., 2022a; Xie et al., 2022). Melatonin triggers the metabolism of most hormones, *e*.*g*., melatonin increases ABA, H_2_O_2_, and ethylene content, participates in signaling molecules, and coordinates biochemical and developmental pathways that change the texture and nutritional quality (Xie et al., 2022). In apple, grape, tomato, and blackberry (*Rubus fruticosus*), the content of endogenous melatonin is changeable and accumulates the most in the flesh during the rapid growth phase, which coincides with the change trend of ethylene in the skin, suggesting its role in regulating phytohormone synthesis (Verde et al., 2022). Furthermore, melatonin-treated fruits have higher quality, number (*e*.*g*., higher 6.6% in blackberry), weight (*e*.*g*., higher 6.6% in grapes and 47.8% in pears), and size (Liu et al., 2019; Verde et al., 2022). Therefore, melatonin does have superior effects on all aspects of fruit development.

#### Post-management of fruit

After maturation, the occurrences of chilling injury, decay, moths, and microbes are the major limiting factors for post-management of the fruit, while melatonin has a unique superiority in maintaining fruits with good storability and quality (Xie et al., 2022). The present studies depict that, in banana, apple or pear, the exogenous application of melatonin distinctly reduces ethylene production during postharvest (Liu et al., 2019; Verde et al., 2022). Delay of senescence in cold-stored mangoes and tomato fruit by exogenous melatonin is attributed to the postponement of the climactic peak of ethylene due to a higher expression of biosynthesis-related genes (*SlACS4*), ethylene receptor genes (*SlNR* and *SlETR4*), and ethylene signaling-related genes (Sun et al., 2020; Dong J. X. et al., 2021). Additionally, during cold storage, melatonin reduces water runoff to regulate ethylene release (Anna and Petriccione, 2022). This view is justified based on two pieces of evidence: one is that melatonin-based coatings influence the expression of several genes, such as wax synthesis genes (*CER1*), cutin monomer genes (*GPAT4/8*), and aquaporin genes (*PIP1;4*, *PIP2;7*, and *PIP22*), which all determine the formation of a surface barrier composed of cuticle to reduce water outflow (Miranda et al., 2020; Anna and Petriccione, 2022). Another is that melatonin benefits metabolic processes (respiration and transpiration), which help to reduce the water vapor pressure gradient between the fruit and the surrounding atmosphere (Rastegar et al., 2020).

Fruits, such as mango and guava, treated with melatonin can maintain significantly higher unsaturated fatty acid levels and higher activities of enzymes (cytochrome c oxidase, H-ATPase, and Ca-ATPase) for the sake of a constant energy supply (Dong et al., 2021; Renu et al., 2022). Crucial enzymes of other processes activated by melatonin, such as the lipid metabolic pathway (*LPS*, *LOX*, and *PLD*), phenylpropanoid metabolic pathway (*4CL* and *PAL*), and shikimic acid pathway (*P5CR*, *P5CS*, and *OAT*), attenuate phosphoinositide and chlorophyll degradation or promote polyamine (PA) accumulation to aid in the fluidity and function of the cell membrane, particularly chloroplast membrane integrity (Aghdam et al., 2019; Silin et al., 2022; Renu et al., 2022). On the other hand, melatonin treatment improves the activities of antioxidant enzymes such as ascorbate peroxidase, glutathione S-transferase, and phenylalanine ammonia-lyase and upregulates genes coding for catalase, manganese superoxide dismutase, copper–zinc superoxide dismutase, monodehydroascorbate reductase, dehydroascorbate reductase, and glutathione reductase, which all decrease ROS, H_2_O_2_, and MDA production and polyphenol oxidase and lipoxygenase activities, resulting in the suppression of mildew and the visual symptoms (pitting, blackening, wrinkling, and browning) that are caused by microorganisms, insects, and brown pigments (Dong J. X. et al., 2021; Ze et al., 2021).

## Melatonin functions against abiotic stress

In nature, the environment changes constantly, which is a huge challenge for plants suffering drought, fire, flood, and temperature stress. Melatonin has emerged as a defense potentiator to keep plants adapting by inducing the synthesis of endogenous melatonin or by improving the accumulation of metabolites (Zhang et al., 2022). By analyzing the transcriptional profile, the expression patterns of related genes (*COMT*, *HIOMT*, *TDC*, *T5H*, *SNAT*, and *ASMT*) under various abiotic conditions are shown (Ahn et al., 2021; Xing et al., 2021). Simultaneously, studies have reported that melatonin application modulates a variety of physiological processes to enhance plant tolerance, which is attributed to the increased endogenous melatonin content (Fan et al., 2018; Arnao et al., 2022). The subsequent chapters are organized according to the role of melatonin and related metabolites in determining plant stability under different stresses and how these changes occurred.

### The effect of melatonin on photosynthesis

It is well known that plants gain energy through photosynthesis due to the sensitivity of chloroplasts to abiotic stresses; thus, the function of the photosystem and production yield are severely limited (Yang S. J. et al., 2021). Interestingly, the occurrence of photosynthesis and melatonin biosynthesis in the same organelle shows a complex connection, which is considered a natural self-protection strategy in plants to ensure stable photosynthesis and maximal food production under unstable environmental conditions (Marino et al., 2022). This result is supported by the phenotype of *Arabidopsis* overexpressing *ASMT9*, showing strong resistance to stress, in which melatonin concentrations, photosynthetic rate, fresh biomass, and dry biomass are significantly increased (Kang et al., 2010). Simultaneously, *COMT1* knockout *Arabidopsis* exhibits reduced photosynthesis, carbon fixation, energy absorption and distribution, and heat stress reactions (Ahammed et al., 2018).

Melatonin has shown excellent protective effects on the integrity of the photosystem (Zhang et al., 2022)—for example, high salinity causes injury to the D1 subunit of PSII, but melatonin application is accompanied by a decline in the content of 34-kDa PSII reaction center protein (D1) and an increase in the content of PSII subunit S protein, which helps tomato and maize achieve high photosynthetic efficiency (Zho X. et al., 2016). Interestingly, melatonin reduces the photosystem protein levels under normal light but increases the PSII and PSI protein contents to cure photosystem damages under high light conditions (Yang S. J. et al., 2021). Melatonin is essential in maintaining a delicate balance between the breakdown and the synthesis of chlorophyll in plants (Khan et al., 2019). A comprehensive analysis of current results shows that melatonin protects chlorophyll from breakdown under stress in several ways, including the acceleration of *de novo* synthesis of chlorophyll or alleviation of chlorophyll loss (Yang S. J. et al., 2021). As mentioned above, those processes involve the expression of chlorophyll degradation-associated (*NYC1*, *NOL*, *CLH*, *PPH*, and *PAO* in melatonin-treated broccoli) and synthesis-associated genes (*POR*, *CAO*, and *CHL G* in tomato) as well as the accumulation of ABA and jasmonic acid (JA) to embody the renewal and stability of chlorophyll in the long term (Wu et al., 2021; Li et al., 2021). Melatonin substantially improves energy flux, which consists of absorption (ABS/RC), trapping energy (TRo/RC), and electron transport (ETo/RC), helping two *Brassica napus* cultivars reduce the toxic effects of polymetals (Ayyaz et al., 2021). Furthermore, in *SNAT1*-deficient *Arabidopsis*, the gene expression of chloroplast heat shock proteins (*CpHSP70.1* and *CpHSP70.2*) and caseinolytic proteases (*ClpR1*, *ClpR4*, and *ClpP1*) is suppressed, while exogenous melatonin application reverses this response, implying that melatonin might participate in the quality control of chloroplast proteins (Lee and Back, 2018). Under the combined stress of low temperature and high humidity, melatonin pretreatment promotes the uptake and translocation of N, Mg, and Fe, which are responsible for chlorophyll biosynthesis and other physicochemical reactions (Amin et al., 2022). Evidence indicates that, when tomato is exposed to darkness for 4 days, melatonin increases the chlorophyll content, Fv/Fm value, and starch content and ameliorates carbon starvation-induced leaf chlorosis by activating the expression of miR171b or inhibiting glucan water dikinase gene expression (Wang et al., 2022b). Moreover, melatonin is involved in the process of the exchange between gas and water by changing the area and the density of stomata (Yang X. X. et al., 2022). Collectively, with the help of melatonin, photosynthetic mechanisms run smoothly, and cooperation efficiency and effectiveness are on the rise to adapt to ensuing changes under environmental stress.

### Enhanced antioxidant system by melatonin

Studies have shown that melatonin is superior in equalizing the production and scavenging of ROS and reactive nitrogen species (RNS) (Lee and Back, 2022; Zhang et al., 2022). The mechanisms underlying the alleviation effects of melatonin on oxidative stress mainly involve crosstalk among various defensive response pathways (Zhang et al., 2022). One of the most effective ways to eliminate reactive oxygen species is to synthesize melatonin as a result of the enhanced antioxidant system (Zhang et al., 2022). In indirect ways, melatonin modulates antioxidant enzyme activities to improve their efficiency in ROS (H_2_O_2_ and O^2−^) detoxification. A series of antioxidant enzymes, POD, APX, SOD, and CAT, are induced by exogenous melatonin to dispose of excessive ROS accumulation (Zeng et al., 2022). Several studies have proposed that, under various abiotic stresses, the melatonin-mediated ascorbate–glutathione (AsA–GSH) cycle serves a crucial role in scavenging reactive oxygen species in cells (Xie et al., 2022; Zhang et al., 2022). Specifically, melatonin enhances the AsA/DHA and GSH/GSSG ratios and antioxidant capacity by stimulating the AsA–GSH cycle, thereby improving the scavenging capacity of ­O_2_·^−^ and ­H_2_O_2_, reducing oxidative stress and providing an approach to reduce organic residues through plant detoxification mechanisms (Dong et al., 2018; Hodžić et al., 2021; Yan et al., 2022). In addition, melatonin could make a direct connection with various oxidizing agents, particularly with hydroxyl radicals, to reduce the damage to cell structures (one molecule of melatonin neutralizes a maximum of 10 molecules of ROS/RNS) (Simlat et al., 2018; Kyoungwhan, 2021). This evidence ties in neatly with the identity of melatonin as a direct antioxidant.

Transcription factors (TFs) are indispensable for regulating gene expression and cover stress-induced biological processes and related metabolic pathways that include cellular processes and primary and secondary metabolism (Xie et al., 2022). In cellular processes, melatonin-mediated gene expression requires TFs to further modulate metabolic processes during the aging of various seeds and maintain higher antioxidant enzyme activities than aging treatment alone (Biswojit et al., 2020). In particular, TFs such as WRKY, AP2/ERF-ERF, MYB, NAC, bZIP, and bHLH have a larger impact (Biswojit et al., 2020; Shi et al., 2015a; Zhao et al., 2021). Wei et al. (2018) confirmed that melatonin synthesis enzymes (*MeTDC2* and *MeASMT2/3*) interact with *MeWRKY20/75* to form a protein complex that extends the complex signaling of melatonin modulation. Not only ROS reduction in PSII but also circadian rhythm fluctuations have been mediated in melatonin-treated tomato, according to the weakened interaction between Sl*CV* and Sl*PsbO*/Sl*CAT3*/Sl*M3H* (Yu et al., 2022). In addition, a set of miRNAs (such as miR8029-3p, miR159-5p, miR858, and novel-m0048-3p) is able to negatively regulate target mRNAs in response to melatonin-mediated cold resistance (Li et al., 2016). Biswojit et al. (2020) observed the noticeable expression of secondary metabolite genes in the transcriptome profile. Similarly, phenolic compounds, flavonoids, and other representative nonenzymatic antioxidant compounds accumulate to control ROS levels *via* the NO-dependent pathway (Kim et al., 2010) and carotenoids (Sharma et al., 2020),

Carbohydrates/fatty acids and amino acids are well known to work as osmoprotectants and ROS scavengers (Marino et al., 2022). Iqbal et al. (2021) emphasized that melatonin initiates carbohydrate metabolism to resist oxidative stress by regulating related genes and repairing damaged membranes and proteins. A correlative study indicated that melatonin induces HSPs and autophagy and reduces oxidized proteins to promote cellular protein protection for the purpose of surmounting biological hurdles (Shi et al., 2015a; Xu et al., 2016). Furthermore, tobacco BY-2 cells, throughout the culture period, are able to synthesize starch by absorbing exogenous melatonin in large quantities to counteract stress-induced damage (Kobylińska et al., 2018). Several reports have shown that melatonin induces higher levels of 18 metabolites (such as cellobiose, galactose, and gentiobiose), 10 amino acids, five sugars (arabinose, mannose, maltose, glucopyranose, and turanose), five polyalcohols (dulcitol, galactinol, glycerol, myo-inositol, and sorbitol), one organic acid (propanoic acid), two sugar alcohols of the carbon metabolic pathway, and more expressed genes related to carbohydrate transcripts, including glycosyl-transferases, glycosyl-hydrolases, glycosyl-phosphatases, glycosyl-invertases, and glycosyl-mutases, hexokinases, mannosidases, α- and β-amylases, α- and β-glucan related-enzymes and several dehydrogenases (3-phosphoglycerate-, UDP-glucose-, alcohol- and aldehyde-), among others (Fan et al., 2015; Hernández-Ruiz et al., 2021). Specifically, in seeds, amid abiotic stress, plants generate important compatible solutes (such as starch and sucrose) to improve osmosis by advancing the expression of pectinesterase, malZ, sucrose-phosphate synthase, glgC, and PYG with the support of melatonin (Su et al., 2018). As previously described, it has been clearly demonstrated that melatonin mediates carbohydrate, polyalcohol, and other metabolite levels in response to osmoregulatory adaptation.

### Melatonin-regulated signaling pathways in response to stress

Signal molecules significantly activate antioxidant responses when plants confront various environmental stress factors (Zhang et al., 2022). First, the regulation of defense-related gene expression, stomatal movement, root morphogenesis, and germination is evidently based on melatonin/NO-mediated signal cascades involving ROS, mitogen-activated protein kinase (MAPK), phytohormones, protein kinases (cyclic adenosine diphosphate ribose and cyclic guanosine monophosphate), and second messengers such as Ca^2+^ and cyclic guanosine monophosphate (Zhu et al., 2019). On the one hand, the multiple effects of NO and melatonin depend on their concentrations (Zhu et al., 2019)—for example, NO can regulate melatonin-related biosynthetic enzymes by forming N-nitrosomelatonin (a kind of NO donor *in vitro* as well) to promote melatonin production (Kaur and Bhatla, 2016). Melatonin improves nitrate reductase (NR) and NO synthase activities and related gene expression to regulate NO production, which further affects a variety of systems (Pardo-Hernández et al., 2020). In rapeseed under saline conditions, a signal cascade of NR and NO associated-1 is triggered by melatonin and NO in a concentration-dependent manner and influences the intensification of melatonin-induced S-nitrosylation that decreases because of the removal of NO (Zhao et al., 2018). Nitrosylation and NO_2_-Tyr are both NO-mediated posttranslational modifications that are related to the activities of some antioxidant proteins, thus helping to maintain the antioxidant capacity (Pardo-Hernández et al., 2020). Inextricably, melatonin stimulates endogenous NO content with a downregulation of s-nitroglutathione reductase expression in tomato seedlings, and an increased expression of glutathione S-transferase genes and AsA/GSH cycle genes (*GR*, *APX*, *DHAR*, and *MDAR*) in kiwifruit makes melatonin play a protective role in defending against damage by regulating antioxidant pathways (Liang et al., 2018; Xie et al., 2022). However, NO and melatonin interact in complex ways (Zhu et al., 2019). The reaction of compounds (such as serotonin, N-nitroso-melatonin, and sodium nitroprusside) releases NO, which interacts with melatonin to maintain oxidative homeostasis through the modulation of SOD isoforms (Cu/Zn SOD and Mn SOD) and the Na^+/^K^+^ ratio through the reprogramming of sodium hydrogen exchanger and salt overly sensitive 2 expression (Zhu et al., 2019). In *Haematococcus pluvialis*, the induction of melatonin enhanced light tolerance and nitrogen starvation resistance by activating the NO-dependent MAPK signal cascade (Zhu et al., 2019). Alleviation of salt-induced damage was also achieved effectively with the correlation factor of melatonin and nitric oxide signaling pathways and the accumulation of ­ONOO− (peroxynitrite anion) and ­O^2^·− (Arora and Bhatla, 2017).

Hormone signal transduction is essential for inducing biological processes and responses to environmental factors, which are known to activate gene transcription and regulate downstream metabolic processes (Su et al., 2018). Typically, melatonin helps to acclimate to thermal shock not only through the differential regulation of HSPs (*HSFB3*, *HSFA1a*, *HSFA2b*, *HSP23*, *HSP70*, *HSP80*, and *HSP90*) but also stimulates Ca^2+^ and hormone signal transduction to accelerate a general response to improve resistance (Xing et al., 2021). Eight subpathways (*viz*., IAA, ABA, CTK, GA, ETH, BR, JA, and SA) were also found to have genetic responses and were analyzed by Zhao et al. (2021)—for example, in the JA synthesis pathway, melatonin significantly regulates the transcripts of nine α-linolenic acid metabolism-related genes in maize seedling leaves (Zhao et al., 2021). In parallel, Jahan et al. (2021) presents a strong cross-connection among three signaling molecules (melatonin, GA, and ABA) and demonstrates that melatonin treatment repressed heat-induced leaf senescence either directly or indirectly by regulating the GA levels. The role of melatonin in the heat stress signaling response is evident in functioning upstream of H_2_S to reduce oxidative stress and increase antioxidative metabolism (Teng et al., 2022). This may suggest that melatonin traces the endogenous H_2_S-dependent pathway, wherein H^+^-ATPase-energized secondary active transport operates K^+^–Na^+^ homoeostasis (Teng et al., 2022). Siddiqui et al. (2021) refers to the stimuli of melatonin-mediated L-DES activity, endogenous H_2_S content, K^+^, and RWC retention that relies on the synergistic effect of melatonin and H_2_S on NaCl stress tolerance.

Furthermore, in melatonin-induced plants, sugars act as signaling molecules, while they positively or negatively regulate the expression of a variety of genes and enzyme activities of sugar-exporting (source) and sugar-importing (sink) tissues for the optimal synthesis and use of carbon and energy resources (Hernández-Ruiz et al., 2021). Teng et al. (2022) observed that treatment with 100 µM melatonin or high sucrose levels can trigger the downregulated expression of *HXK* and the upregulated expression of *PFK7* so that source-to-sink phloem transport is promoted. Carbon metabolic flux of primary metabolism will be rebalanced as a consequence of elaborate management through these processes (TCA cycle and glycolysis) to meet the metabolism demand in organisms at risk (Teng et al., 2022). It is clear for melatonin-based mechanisms that various signal pathways are important for the integrity and fitness of living organisms.

### Accumulation of secondary metabolites regulated by melatonin

**Figure 2:**
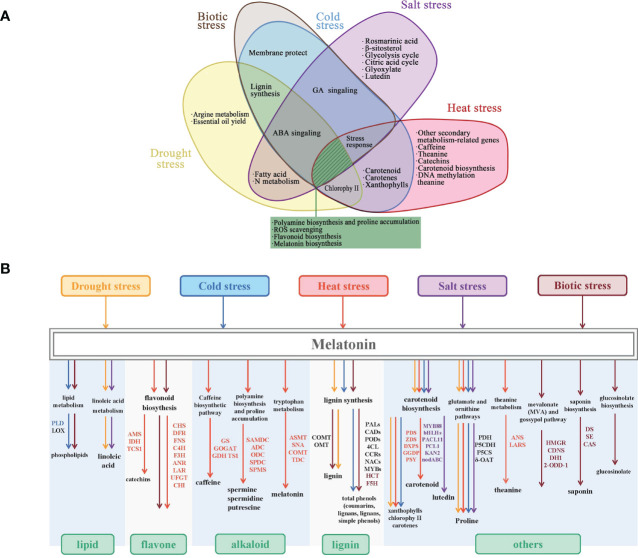
Synthesis and metabolism patterns of melatonin-induced secondary metabolites in response to stress. **(A)** Melatonin modulates various mechanisms against abiotic stresses (salt, cold, heat, and drought). **(B)** Melatonin induces common changes in gene expression in secondary metabolic pathways. PLD, phospholipase D; TCS1, tea caffeine synthase 1; CHS, chalcone synthase; DFR, dihydroflavonol 4-reductase; TIDH, inosine 5′-monophosphate dehydrogenase; LOX, lipoxygenase; AMS, S-adenosyl-l-methio-nine synthase; FNS, flavone synthase; C4H, cinnamic acid 4-hydroxylase; F3H, flavanone 3-hydroxylase; ANR, anthocyanidin reductase; LAR, leucoanthocyanidin 4-reductase; UFGT, UDP-glucose: flavonoid 3-O-glucosyltransferase; CHI, chalcone isomerase; GR, glutathione reductase; GSH, glutathione; GSNOR, S-nitrosoglutathione reductase; TS1, theanine synthase; ASMT, acetylserotonin O-methyltransferase; SNAT, serotonin N-acetyltransferase; COMT, caffeoyl-O-methyltransferase; TDC, tryptophan decarboxylase; OMT1, flavone 3′-O-methyltransferase 1; PAL, phenylalanine ammonia-lyase; CAD, cinnamyl alcohol dehydrogenase; POD, peroxidase; 4CL, 4-coumarate-CoA ligase; CCR, cinnamoyl CoA reductase; NACs, IAA-related factors; MYBs, regulator of CBF; PDS, phytoene desaturase; ZDS, ζ-carotene desaturase; bHLHs, basic helix–loop–helix; PCL1, phenylalanine ammonia lyase 1; PDH, pyruvate dehydrogenase; P5CDH, delta-1-pyrroline-5-carboxylate dehydrogenase; P5CS, pyrroline-5-carboxylate synthetase; OAT, ornithine aminotransferase; F5H, ferulic acid 5-hydroxylase; HCT, hydroxycinnamoyl transferase; HMGR, HMG-CoA reductase; CDNS, (+)-d-cadinene synthase; DH1, alcohol dehydrogenase; 2-ODD-1, 2-oxoglutarate/Fe(II)-dependent dioxygenase; DS, dammarene diol-II synthase; SE, squalene epoxidase; CAS, cyclohexene synthase.

To cope with plant stress signals, various melatonin-regulated metabolic pathways are accompanied by the accumulation of substances (such as flavonoids, polyamines, and phenolic compounds) and the improvement of organelle function (Marino et al., 2022). More attention to physiological and transcriptomic analyses of the effects of exogenous melatonin on stress tolerance has proven this perspective (**Figure 2**).

#### Effects of melatonin on polyamine biosynthesis

PAs, namely, putrescine (Put), spermidine (Spd), and spermine (Spm), are considered a class of important regulators and have been shown to be involved in mitigating deleterious effects when plants suffer (Marino et al., 2022). Studies have demonstrated the association of melatonin and PA metabolism in response to environmental stress, as described in Bermuda grass (Shi et al., 2015b), carrot (Lei et al., 2004), peach (Cao et al., 2016), and *Malus hupehensis* (Gong et al., 2017)—for instance, melatonin, as a resistant initiator under diverse environmental stress conditions, including water shortage, low temperature, salt, and nutrient deficiency, can not only upregulate the synthesis genes (*ODC*, *ADC*, *NCA*, and *SPDS*) and downregulate the degradation genes (*PAO* and *DAO*) of PA but also stimulate the activities of related enzymes, including NR, ADC, ODC, nitrite reductase, glutamine synthetase (GS), glutamate synthase (GOGAT), and S-adenosylmethionine decarboxylase, inducing a significant increase in polyamines and endogenous melatonin content (Zhou C. et al., 2016; Talaat, 2021; Lei et al., 2022; Wang et al., 2022a). In addition, melatonin was found to accelerate the synthesis of amino acids (such as arginine and ornithine), which are precursors of bioactive compounds (such as Spd, Spm, Put, and GABA compounds) (Wang et al., 2022a). Therefore, by accelerating polyamine biosynthesis, melatonin attenuates apoptosis and disintegrates the membrane in cold-induced carrot and tomato (Ding et al., 2017; Aghdam et al., 2019). In melatonin-treated samples, the accumulated arginine and polyamine contents are also beneficial for nitrogen distribution and recycling, modulation of ion channel activities (specific polyamine-binding proteins) and Ca ^2+^ homeostasis, as well as the activation of NO signaling pathways, which are of prime importance to improve the resistance of Fe-deficient plants (Zhou C. et al., 2016; Wang et al., 2022a). Otherwise, the interplay between melatonin and SA, ABA, and ethylene accumulation plays crucial roles in the stress management of polyamines (Zhao et al., 2017). Both ABA and PA contents are increased in the crosstalk of melatonin and ABA, which upregulates an important stress-related gene, *CsZat12*, leading to the mitigation of chilling stress in cucumber (Zhao et al., 2017). However, in water-logged alfalfa, melatonin antagonizes ethylene production by suppressing ethylene biosynthesis-related gene expression and moves to PA biosynthesis (Zhang et al., 2019). Interestingly, melatonin metabolites (2-hydroxymelatonin) provide utility for regulating metabolism, particularly for enhancing Put, Spm, and Spd synthesis, and this exhibits a preferred orientation for studying the mechanism of melatonin to counteract environmental stress (Shah et al., 2020). A profound influence on plant resistance exerted by melatonin is evident herein through the elevated polyamine content, which scavenges free radicals, preserves nucleic acid and protein structures, and consequently improves membrane stability.

#### Effect of melatonin on flavonoid biosynthesis

Flavonoids have a main effect on protecting plants from abiotic stress by acting as a backup for peroxide production to eliminate various types of ROS (Dong et al., 2018). Studies designed with various stress conditions (such as cold, water deficiency, salt, *etc*.) show that melatonin promotes secondary metabolite accumulation, including flavones, flavanone, luteolin, and isoflavone (Ithal and Reddy, 2004; Song et al., 2022). It was also reported that the transcriptional levels of *PCL*, *CHS*, *DFR*, *FNS*, *C4H*, *F3H*, *ANR*, *LAR*, *UFGT*, *CHI*, and *IFS* genes involved in flavonoid biosynthesis were significantly increased by melatonin treatment (Dong et al., 2018; Song et al., 2022). In addition, *PAL* and *PPO* have been shown to be upregulated in *Dracocephalum moldavica*, *Vigna radiata*, and *Sesamum indicum* treated with melatonin, and damage inhibition was observed (Naghizadeh et al., 2019). In heat-treated chrysanthemum and kiwifruit, cold-stressed white clover and kiwifruit, and salt-induced pigeon pea, similar data have also been obtained—that is, melatonin favors the production of flavonoids, carotenoids, carotenes, xanthophylls, and chlorophyll II to protect the photosynthetic potential and mitigate adverse effects (Sun et al., 2016; Liang et al., 2018; Dong et al., 2018; Sun et al., 2020; Xing et al., 2021; Song et al., 2022). Isoflavone biosynthesis responds to melatonin profoundly through hormone biosynthesis involving upregulated genes (*AOS*, *AAO*, *SAMA*, and *ACCO*), which has conferred a broader understanding of the melatonin regulatory mechanism (Gyanendra et al., 2021). Otherwise, it has been verified by Song et al. (2022) that melatonin greatly decreases genomic DNA methylation and modified gene expression. Therefore, the outcome of increased disease resistance and flavonoid biosynthesis is at least partially achieved by utilizing melatonin to decrease the methylation of related gene promoters, such as *EDS1* (Gao et al., 2020). Conversely, in some cases, among gardenia and apples, melatonin decreases the related gene expression levels of flavonoids but with a greater retention of carotenoids so that plants remain greener and have brighter leaves after 24 days in dark conditions. This internal indication illustrated that melatonin-treated plants adapted better to the adverse environment (Wang et al., 2018). Similarly, overexpressing *MsASMT1* in alfalfa makes it grow faster and have higher endogenous melatonin levels, which, in turn, suppress flavonoid biosynthesis (28 downregulated genes), mainly quercetin, kaempferol, formononetin, and biochanin (Cen et al., 2020). Notably, flavonoids are potent in inhibiting *in vivo* melatonin biosynthesis by abolishing the activities of ASMT, SNA, and COMT (Kyungjin et al., 2018). In terms of the objective use of melatonin, some notable changes in flavonoids are used to establish the ideal dose to influence plant resistance to develop effective solutions and to provide a fresh perspective that explores the connection between melatonin and secondary metabolites.

#### Effect of melatonin on other secondary metabolites

Multiple secondary metabolisms are associated with plant stress tolerance, while in many persuasive studies, melatonin application shows an increase in total phenolic content, total flavonoid content, rosmarinic acid, luteolin flavone, apigenin flavone, photosynthetic pigment, ascorbic acid, phenol, and essential oil (EO) yield (Saad and Salem, 2019; Farinaz et al., 2020). Taking phenolic compounds as an example, the levels of resveratrol, caffeic, chlorogenic, and gallic acids can be redistributed by melatonin in stressful situations (Xu et al., 2018). Scientists have clearly demonstrated that melatonin function in moderately high-temperature-stressed tea is accompanied by increased contents of catechins, theanine, and caffeine, which are related to upregulated genes, including *AMS*, *IDH*, *TCS1*, *ANS*, *LAR*, *GS*, *GOGAT*, *GDH*, and *TS1* (Xin et al., 2020).

As a crucial secondary metabolite generated through the phenylalanine metabolic pathway, lignin has practical effects on protective barriers (Wang et al., 2019). Melatonin addition can compensate for the blockaded lignin accumulation and the decreased oxidase activities (such as PAL, CAD, and POD) caused by low temperature. This evidence indicates that, under cold stress, melatonin regulates lignin synthesis genes (*ZlPAL1/2/3/4*, *ZlCAD1/2/3*, and *ZlPOD1/2/3/4/5*) and TF expression (NAC and MYB family) in bamboo and therefore is expected to benefit from horticultural management with melatonin application (Li C. T. et al., 2019). This result is consistent with that of Wang et al. (2021). Additionally, melatonin acts as a positive signal that enhances *P. lactiflora* stem strength and lignin accumulation by regulating the expression of genes (*PAL*, *4CL*, *CCR*, and *CAD*) in lignin biosynthesis (Zhao et al., 2022). Given the resistance of *V. dahliae*, the gossypol and lignin contents in cotton were increased by melatonin through changes in the metabolic flux of different pathways [mevalonate (MVA), phenylpropanoid, and gossypol pathways] (Li Z. et al., 2019).

Other secondary metabolites, such as glucosinolates, are known to defend against herbivores and pathogenic attacks (Selmar, 2010). Some secondary metabolites are relevant to maintaining plant commercial life when florets of broccoli treated with melatonin can raise physical–chemical parameters, including color, texture, shine, and rates of weight loss (Arnao et al., 2022). Saponins and alkaloids, both mediated by melatonin, are beneficial for the self-protection of plants (Ptak et al., 2019; Yang Q. et al., 2021). For chromium-treated rosemary, melatonin applications will increase its yield and EO yield by up to 25% and 100%, respectively (Farouk and Al-Amri, 2019). Moreover, as an active phytosterol, β-sitosterol significantly lowers stress caused by high salt levels in tomato and sunflower (*Helianthus annuus*) and is contained in many plant species, and its density and production are significantly increased in dehydrated plants when exposed to melatonin treatment, thus hinting at the underlying relationship (Fawzia et al., 2016; Gamel et al., 2017; Ramadan et al., 2018).

Further exploration of the role of melatonin may greatly enhance specific secondary metabolite synthesis, thereby improving defense capability and leading to less cell death and disease in living organisms, instead of some stress-induced processes, such as lipid peroxidation.

## Melatonin functions against biotic stress

Melatonin is unique in the mitigation of plant biological stress. First, the identification of melatonin as a scavenger has cleaned not only ROS and RNS but also viruses (Mohamed et al., 2019). Apples infected with apple stem grooving virus shed light on this discovery in such a way that melatonin eradicates the virus from previously infected shoot tips (Mohamed et al., 2019). The second is melatonin-activated bioactivity, such as phagocyte and plant resistance (R) proteins (Mohamed et al., 2019; Rahul et al., 2022). Relatedly, regulated genes are mapped to signal transduction networks (Rahul et al., 2022). Melatonin upregulates defense genes through SA, ethylene, and NO signaling, and another hormonal crosstalk (CKs, ABA, IAA, and GA) also involves melatonin in systemic acquired resistance (Mohamed et al., 2019; Rahul et al., 2022). Additionally, melatonin stimulation induces a response from the MAPK cascade as confirmed in (Pst)-DC3000-infected *Arabidopsis* and SlMPAK3-deficient tomato infected with *Botrytis cineraria*, which also leads to the initial PAMP-triggered immunity response and effector-triggered immunity response (Rahul et al., 2022). Thirdly, plants will gain strengthened physical obstacles in that more complex chemicals are generated, and genes of dammarene diol-II synthase (DS), squalene epoxidase (SE), and cyclohexene synthase in saponin biosynthesis, HMG-CoA reductase (HMGR), (+)-d-cadinene synthase, alcohol dehydrogenase in the MVA and gossypol pathways, and other genes in melatonin, flavonoid, lipid, and lignin biosynthesis are upregulated by melatonin stimuli (Mohamed et al., 2019; Gao et al., 2020; Yang Q. et al., 2021).

## Conclusions

Melatonin has a unique role in every stage of the plant life cycle, particularly in reversing environmental pressure. It is produced in the cytoplasm, chloroplasts, and mitochondria, which may be conducive to easy movement and prompt response. Several interpretations to explain the stress protective function of melatonin in plants have been proposed in this paper and zeroes in on the regulation of primary and secondary metabolism with the objective of capturing its versatile role in the plant hormone network and general physiological processes. At the same time, melatonin regulates a wide range of molecular mechanisms, not only interacting with proteins and affecting nutrient metabolism in plants but also having functional effects on noncoding RNA. This potential gives melatonin priority over pesticides and fertilizers to become a sort of pollution-free solution maintaining the supply for plant-based foods and products. To varying degrees, melatonin and its precursors and metabolites help to enhance plant immunity and development. These data indicate another pioneering work on melatonin pathway application that provides multiple options for breeding work. Overall, the study of the real risks and benefits of many secondary metabolites in different plants is a way to decipher their genetic structures and inherent gifts. It would be a marked progress if we knew how they work with melatonin in such a way that arouses strong resistance toward stresses in unexpected ways.

## Author contributions

YP and JZ: conceptualization. YP and XX: writing of the manuscript and preparation of the figures. LL, QS, QW, HH, ZT, and JZ: revising and editing. All authors contributed to the article and approved the submitted version.
